# Multi-scale mechanical characterization of the lining canvas of *The Night Watch* by Rembrandt

**DOI:** 10.1038/s40494-025-02215-7

**Published:** 2025-12-06

**Authors:** S. Maraghechi, E. Bosco, J.P.M. Hoefnagels, A.S.J. Suiker, K. Keune, P. Noble

**Affiliations:** 1 Department of the Built Environment, Eindhoven University of Technology, Eindhoven, The Netherlands; 2 Department of Mechanical Engineering, Eindhoven University of Technology, Eindhoven, The Netherlands; 3 Department of Conservation & Science, Rijksmuseum, Amsterdam, The Netherlands; 4 Van’t Hoff Institute for Molecular Sciences, University of Amsterdam, Amsterdam, The Netherlands

## Abstract

This contribution presents a multi-scale experimental analysis of the mechanical properties of the lining canvas of *The Night Watch* (1642), a large-format canvas painting by Rembrandt van Rijn. The original canvas has deteriorated significantly, leaving the lining canvas as the primary load-bearing component. Mechanical properties, including Young’s modulus, tensile strength, and strain at fracture, are evaluated at three hierarchical levels (fibre, thread, canvas) using advanced in-situ tensile testing combined with optical profilometry, optical microscopy, and Digital Image Correlation. The study distinguishes between the *composite* properties of the lining canvas, reflecting the combined behaviour of fibres, wax resin, and voids, and the *intrinsic* properties, representing the fibre material alone. Scaling laws are established to relate stiffness and tensile strength to the characteristic material length scale, enabling predictions of canvas-scale behaviour from non-invasive fibre-level analyses. These findings may inform future conservation strategies for the canvas of *The Night Watch* and other paintings.

## Introduction

**Fig. 1:**
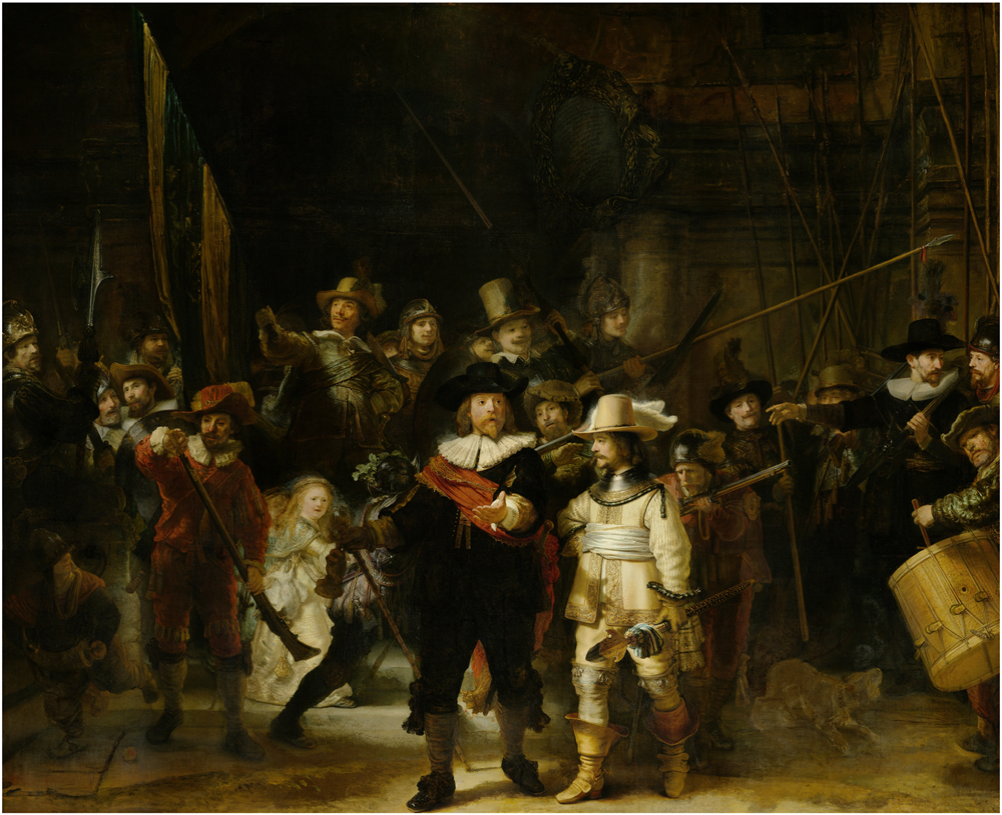
The Night Watch by Rembrandt van Rijn. “The officers and civic guards of District II of Amsterdam under the command of Captain Frans Banninck Cocq and Lieutenant Willem van Ruytenburch”, known as *The Night Watch*, by Rembrandt van Rijn (1642). Canvas, 363 x 438 cm, Rijksmuseum, Amsterdam, The Netherlands, on loan from the City of Amsterdam.

*The Night Watch* (1642), a large-format canvas painting by Rembrandt van Rijn (1606–1669) displayed at the Rijksmuseum, Amsterdam, the Netherlands, is one of the most renowned masterpieces of the seventeenth century (Fig. 1). Over the centuries, this work of art has undergone several conservation interventions, including structural repairs of old damages (holes, tears, etc.) and three wax-resin linings in 1851, 1945 and 1975–76. As the original canvas has significantly deteriorated and no longer provides structural support, the lining canvas now essentially bears the mechanical load. Understanding its properties and state of degradation is therefore crucial to guarantee the painting’s structural stability and to inform effective conservation strategies.

**Fig. 2:**
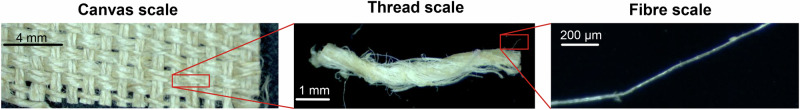
Multi-scale structure of the 1975 lining canvas. Multi-scale structure of the 1975 lining canvas. Macroscopic canvas scale (left), thread scale (centre), and individual fibre scale (right).

The mechanical behaviour of a canvas is governed by its multi-scale structure, which spans across different length scales (Fig. 2). Individual linen fibres, composed of cellulosic flax, are aligned and twisted together to form threads. These threads are then organised into a woven structure, with warp threads running longitudinally and weft threads interlaced perpendicular to them. This configuration defines the interwoven geometry of the canvas at the macroscopic level. Moreover, additional materials, such as the wax-resin mixture used to attach the lining canvas to the original canvas, further influence its mechanical behaviour. This hierarchical complexity and material heterogeneity require a multi-scale approach to fully capture the canvas’s mechanical response.

Existing studies have primarily addressed the macroscopic mechanical properties of mockup canvas samples, typically analysed using standard uniaxial^1–3^ and biaxial^1,2,4^ tensile tests. Tensile tests have also been employed to examine the effects of environmentally-induced ageing^5–8^ and lining adhesives^9,10^ on the mechanical response of canvases. Advanced optical methods, including fibre Bragg grating^11^ and full-field DIC (Digital Image Correlation)^12–14^, have provided additional insights into the deformation behaviour of painted canvases and historical textiles. However, these techniques do not directly measure mechanical properties, which limits their applicability in understanding the structural response of canvases. At the thread level, no direct mechanical measurements specific to painting canvases are currently available. Related studies from other fields include uniaxial tensile tests of cellulose-based threads and yarns used in historical tapestries^15^, sewing threads^16^, woolen textiles^17^, and technical textiles^18,19^. At the fibre scale, micro-tensile testing has been applied to a wide range of fibres, such as organically-formed cellulose fibres^20–24^, regenerated cellulose fibres^24–27^, flax fibres^18,19^, and wood fibres^28–30^. While these studies demonstrate the versatility of micro-tensile tests, they primarily focus on modern or technical applications rather than heritage materials. In summary, current studies reveal three main limitations: (i) a primary focus on single-scale analyses, without correlation of data across different scales for canvases used in historic paintings; (ii) limited integration of advanced full-field deformation measurement techniques with mechanical testing in the field of cultural heritage; (iii) a predominant use of model systems, largely due to the limited availability or impracticality of testing original historical materials.

The present contribution addresses these critical gaps by: (i) proposing a multi-scale methodology to analyse and correlate the mechanical properties (namely Young’s modulus, ultimate tensile strength, and strain at fracture) of canvas samples at the fibre, thread, and canvas scales; (ii) integrating mechanical testing with advanced in-situ observations using optical microscopy and optical profilometry combined with DIC; and (iii) developing a framework that enables estimation of macro-scale material properties through minimally invasive analysis of original canvas materials at smaller scales, thus avoiding the exclusive use of mock-ups.

Although demonstrated here on the lining canvas of *The Night Watch*, the methodology is broadly applicable to the canvas supports of other historical paintings and can serve as a valuable tool for future conservation strategies.

This paper is organised as follows. Section “Methods” describes the canvas support of *The Night Watch* and the methodologies used to assess the mechanical properties of the lining canvas and a reference canvas at fibre, thread, and canvas scales. The reference canvas serves as a control sample, obtained from unused canvas material from the 1975 relining treatment. The experimentally measured quantities correspond to *composite* properties, which represent the overall mechanical behaviour of the lining canvas as a structure composed of fibres, wax resin, and voids. In the same section, correction factors are introduced and computed to obtain the corresponding *intrinsic* properties, isolating the load-bearing role of the cellulosic fibres by accounting for porosity, wax-resin content, and geometric irregularities. Section “Results” presents the experimental results, detailing the *composite* and *intrinsic* mechanical responses at each scale. These results are analysed across length scales to establish scaling laws that relate the measured mechanical properties to the characteristic length scale of the material. These scaling laws may enable estimation of canvas-scale behaviour from minimally invasive fibre-scale tests, reducing the need for destructive sampling—an essential advantage given the limited availability of historical canvas material. A statistical comparison with the reference canvas provides further insight into the degradation of the lining canvas. Finally, the main conclusions are summarised in Section 4.

## Methods

### Characteristics of the canvas support of *The Night Watch*

The original canvas of *The Night Watch* is a medium-weight, plain-weave linen fabric, which consists of three horizontal strips stitched together to form a large surface measuring 363 cm × 438 cm. On average, the upper strip contains 12.2 vertical and 12.9 horizontal threads/cm; the central strip, 12.5 vertical and 12.4 horizontal threads/cm; and the bottom strip, 12.1 vertical and 12.8 horizontal threads/cm^31^. Originally larger (approximately 400 cm × 500 cm), the canvas was trimmed during earlier conservation interventions, reducing its overall dimensions. The seams between the three horizontal strips represent structural weak points, and splits along these seams are also visible in the X-radiograph of the painting. The average thickness of the original canvas is approximately 0.5 mm^32^.

**Fig. 3:**
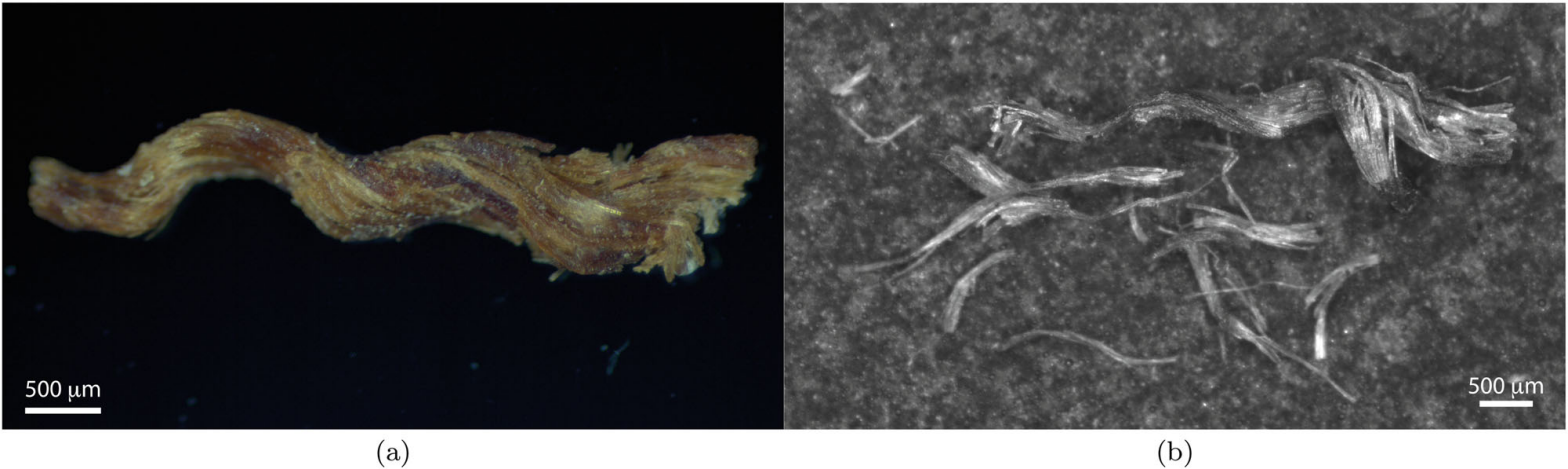
Original canvas. Optical microscopy images of a thread sample from the original canvas of *The Night Watch*. **a** The thread before fibre extraction attempts. **b** The thread after fibre extraction attempt, showing broken fibres that illustrate the extreme brittleness and advanced degradation of the canvas material.

Currently, the back of the original canvas of *The Night Watch* is covered by the lining canvas, which restricts the collection of samples for inspection or measurement. However, fragments from the original canvas became available due to damage incurred by vandalism in 1975. One of these threads, with a length of approximately 4.7 mm, was examined to assess the condition of the original canvas (Fig. 3a).

Attempts to extract fibres from the thread for characterisation were unsuccessful. The fibres of the original canvas were found to be extremely brittle; even the slightest manipulation with fine-tip tweezers under a stereo microscope caused them to fracture into small pieces, as shown in Fig. 3b. This brittleness made it impossible to prepare fibre specimens using the methods outlined in Section “In-situ tensile tests on fibre specimens” and detailed in ref. ^24^.

To investigate whether increasing the moisture content in the original fibres might facilitate their extraction, an additional attempt was made by subjecting the thread to 81% relative humidity (RH) at room temperature for two days prior to extraction. However, the fibres remained brittle and fractured under minimal handling. This pronounced brittleness indicates advanced degradation of the original canvas, suggesting that its contribution to the overall load-bearing function of the painting is negligible. In contrast, the lining canvas provides the primary structural support, and the testing methodology was therefore applied exclusively to this material.

The canvas of *The Night Watch* has undergone numerous conservation treatments throughout its history, beginning at least as early as 1697 when boiled oil was applied to the back of the painting. In 1851, the canvas was wax-resin lined for the first time, although detailed records of this treatment are not available. Between 1946 and 1947, following World War II, the painting was relined again to address deformations in the canvas support. This treatment involved the application of a thick lining canvas, adhered with a wax-resin mixture composed of beeswax, resin, and Venice turpentine in a 5:4:1 ratio. However, because the thick lining canvas absorbed much of the adhesive, the adhesion was insufficient, particularly near the centre of the painting^33^.

The most recent lining canvas, applied in 1975–1976 after the painting was slashed twelve times with a serrated knife (including several cuts approaching one metre in length across the centre), remains in place today. This canvas is a coarser, denser basket-weave linen (two threads over two threads) than the original, with 19 threads/cm in the warp and 17 threads/cm in the weft, made from a single, seamless piece of linen. To attach this to the reverse of the painting, a molten mixture of beeswax and colophony (5:2 ratio) was brushed onto the surface, and then ironed, promoting better penetration and adhesion between the original and lining canvases^33,34^.

Mechanical tests were conducted on both the wax-resin-treated lining canvas from *The Night Watch* and a control sample taken from unused linen from the 1975 treatment. This control sample, stored in the dark and free of wax resin, serves as a reference for comparison. Hereafter, the actual lining canvas from *The Night Watch* is referred to as the *lining canvas* and the untreated control sample as the *reference canvas*.

### In-situ tensile tests on fibre specimens

The mechanical properties of the fibres were evaluated using a recently developed method combining in-situ micro-tensile tests with optical profilometry. In this work, the term in-situ is used in the sense commonly adopted in experimental mechanics, referring to tests performed within an observation instrument (e.g., the optical profilometer) rather than to direct measurements on the painting itself. The complete methodology is detailed in^24^ and is summarised below.

### Sample preparation and cross-sectional area evaluation

First, individual fibres were extracted from a loose canvas thread under a stereo microscope and cut into segments approximately 2 mm in length. Each fibre was then gently brushed with a fine-tip paintbrush to remove any wax-resin residues and to ensure it was straight. Subsequently, the fibre was mounted and secured at both ends to a rigid PMMA (polymethylmethacrylate) frame. The frame had a central window defining an ≈ 800 μm gauge length and two ≈ 500 μm side bridges. Both fibre ends were fixed to the frame with nail polish to preserve integrity before testing.

Prior to the test, surface height profiles of both the top and bottom surfaces were recorded using White Light Interferometry (Bruker NPFLEX, 20 × objective lens). The bottom surface was measured via reflection from a mirror, allowing full 3D reconstruction of the fibre’s shape and cross-sectional area. A speckle pattern was next applied to the top surface of the fibre to enable accurate strain measurement during the test using DIC. This pattern was created by spraying spherical polystyrene particles with a diameter of 1 μm, diluted in ethanol, from a distance of 6 cm for approximately 10 min using an airbrush operated at 1 bar pressure. To prevent irreversible deformation or damage during handling, the frame-fibre assembly was clamped in the tensile stage; immediately before loading, the side bridges were locally melted with a heated wire to transfer the full load to the fibre.

### Micro-tensile test

Micro-tensile tests on the fibre specimens were conducted using a Kammrath & Weiss micro-tensile stage equipped with a 20 N load cell. White Light Interferometry at higher magnification, conducted with the same Bruker NPFLEX system with a 100 × objective lens, was employed to capture the height profiles of the specimen’s top surface along its entire length at each load step, enabling high-resolution strain field measurements. The tests were performed under displacement control, with incremental displacements of 5 μm applied at a rate of 0.5 μm/s. Optical profilometry images were acquired at each load step, with acquisition times ranging from 5 to 10 min. During testing, the local stress at designated Regions of Interest (ROIs) along the fibre was incrementally computed from the applied force and the corresponding local cross-sectional area. Local strain fields along the fibre length were determined at the same ROIs from the height profiles acquired during micro-tensile testing, which were spatially correlated using a dedicated Global Digital Height Correlation (GDHC) algorithm. GDHC is a full-field displacement measurement technique that compares height profiles of a specific ROI on the specimen’s surface taken before and after the application of a load increment. As a modified form of DIC, GDHC is particularly suited to capture stress in the presence of out-of-plane displacements, such as curvature and twisting, which are commonly observed during tensile tests of irregular natural fibres^24^.

**Fig. 4:**
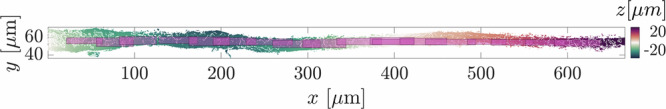
Fibre scale. Height profile of the top surface of a single linen fibre extracted from the lining canvas of *The Night Watch*, acquired by optical profilometry. The magenta rectangles represent the ROIs over which the local strains and stresses are evaluated.

For each load increment, GDHC tracked the displacement of individual material points on the fibre’s surface in all three spatial directions by comparing the positions of corresponding pixels in the original and deformed height profiles. From these displacements, the displacement field within each defined ROI was expressed as a linear combination of second-order polynomial basis functions weighted by a set of initially unknown degrees of freedom. The optimal displacement field was obtained by minimizing the least-squares norm of the residual field with respect to the measured displacements. Once the parameterized three-dimensional displacement field was determined, the surface strain tensor was computed relative to the non-flat initial surface by calculating the logarithmic strains in the two in-plane directions, *x* and *y*, from the corresponding stretch ratios. After computing the surface strain fields for all load steps, the average surface strain in the loading direction was evaluated within each ROI at each increment and subsequently used to construct the local stress-strain response for that ROI. This method typically produced 10–20 stress-strain curves per fibre. Assuming that material and morphological variations along a single fibre are comparable to those between different fibres, the local stress-strain data collected from an individual fibre can be used to statistically estimate representative mechanical parameters from a limited number of tensile tests. Furthermore, the ultimate tensile strength of each fibre was determined by dividing the maximum load applied by the minimum cross-sectional area of the fibre. Figure 4 shows the height profile of the top surface of one of the tested fibres, with all the ROIs depicted by overlaying magenta rectangles.

### In-situ tensile tests on thread specimens

The mechanical properties of the thread specimens from the lining canvas of *The Night Watch* and the reference canvas were characterised using in-situ micro-tensile testing combined with optical microscopy.

### Sample preparation and cross-sectional area evaluation

**Fig. 5:**
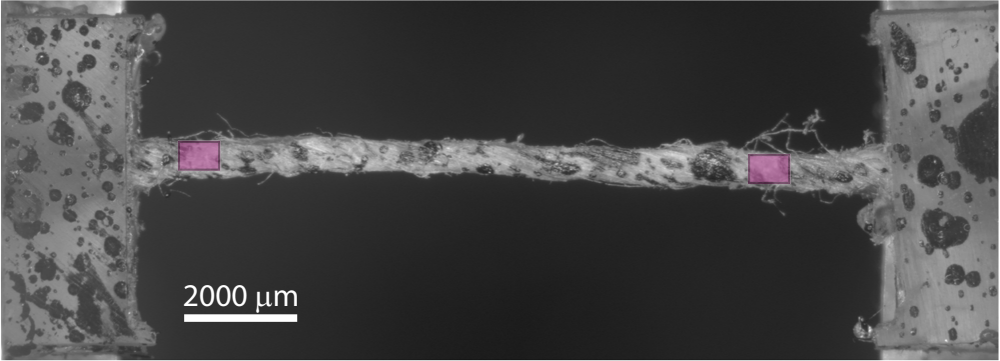
Thread scale. Optical microscopy image showing the top view of a single warp-thread specimen from the lining canvas of *The Night Watch*. The black spots indicate the applied speckle pattern used for DIC. The PMMA slabs clamping the specimen are partially visible along the image edges. The two magenta rectangles correspond to the ROIs selected for evaluating the global displacement of the specimen using DIC.

Thread specimens for the experiment were prepared by extracting single threads from the lining canvas and cutting them into segments approximately 25 mm long. The end parts of each thread were gently flattened and positioned on two 0.5 mm thick PMMA slabs, defining a gauge length of 800 μm, leaving approximately 10 mm of redundant thread loosely hanging between the slabs. The threads were fixed to the slabs using nail polish, after which two additional PMMA slabs were placed on top, forming a sandwich structure that securely enclosed the nail polish-saturated ends. Each specimen was then placed under a heavy weight overnight to ensure optimal grip of the threads after the glue had cured. Next, a speckle pattern for DIC was applied to the top surface of the specimens by carefully splashing black India ink onto the specimen under a stereo microscope using a short-hair paintbrush held at a distance of roughly 5 cm. Before tensile testing, the cross-sectional area of the threads was estimated assuming an elliptical cross-section. Top and side images of each specimen were acquired to measure local width and thickness along the full gauge length, excluding a small region near the grips where the threads were partially flattened. The mean values of width and thickness were taken as the major and minor axes of the ellipse, from which the average cross-sectional area of the thread was calculated. Figure 5 shows a top view of a warp-thread specimen clamped between two PMMA slabs. The black spots correspond to the applied speckle pattern, while the two magenta rectangles indicate the ROIs used to evaluate the global displacement of the specimen by Global Digital Image Correlation (GDIC).

### Micro-tensile test

After preparing the thread specimens, the in-situ tensile test was performed using a Kammrath & Weiss micro-tensile stage with a 100 N load cell. Image acquisition was performed via optical microscopy using a ZEISS Discovery V.20 stereo microscope. Optical microscopy was selected on the thread scale because of its higher measurement efficiency compared to profilometry, even though it only captures two-dimensional surface displacements. For each specimen, the PMMA slabs were securely clamped in the grips of the micro-tensile stage to prevent slippage during loading. The side bridges in the PMMA frame kept the specimen fixed and undamaged during preparation; these side bridges were then carefully removed immediately before loading, following the same procedure used for the fibre-scale tests. The tensile test was conducted continuously under displacement control at a rate of 2 μm/s, with images captured every 5 s. Loading and image acquisition continued until specimen failure.

The threads exhibit a complex microstructure composed of spun fibres, which produces local twisting and out-of-plane motion during loading. These effects, combined with the two-dimensional imaging limitation of optical microscopy, make local speckle tracking unreliable along the entire gauge length. For this reason, a global strain evaluation was adopted. This approach is acceptable considering that out-of-plane deformations at the thread scale, relative to the specimen size, are moderate compared to those at the fibre level, and less variation of strain is expected along the thread length. The global strain was determined using GDIC by first computing the average displacement within two ROIs near the specimen ends (magenta rectangles in Fig. 5), and next dividing the relative displacement between the ROI centres by the gauge length. The ROIs were positioned close to the specimen ends to capture an accurate average gauge length, while maintaining a small distance from the glued areas to avoid boundary effects. The relatively large speckle pattern applied on the thread samples proved to be adequate for reliable tracking of rigid-body motion using GDIC, given the first-order polynomial parametrization of the displacement field.

The average stress at each load step was calculated by dividing the axial force measured by the load cell by the average cross-sectional area along the length of the thread. The resulting average stress values were then paired with the corresponding average strain from GDIC to construct the stress-strain curve of each thread specimen, from which the Young’s modulus, strain at fracture, and ultimate tensile strength were determined.

### Correction factors for cross-sectional area

To determine the *intrinsic* mechanical properties at the thread scale, the cross-sectional area of the thread specimens was corrected to reflect only the load-bearing contribution of the cellulosic fibres. Three correction factors were applied: two account for porosity - area contraction during loading and fibre packing density - and one excludes the wax-resin fraction from the total solid phase (comprising both wax resin and fibre). The corrected areas are used in the results (Section “Thread scale”), while the calculation of each correction factor is described below.

- *Contraction correction factor*. The contraction correction factor accounts for the reduction in the cross-sectional area under axial tension. As tension increases, the thread’s helical microstructure tightens, causing the fibres to twist and compact, which progressively reduces the cross-section until failure. Assuming an elliptical cross-section with constant aspect ratio, this effect was quantified by measuring the specimen width (major axis) at each load step from top-view microscopy images along its length and computing a length-averaged value. The corresponding cross-sectional area at each load step was then derived, as shown in Fig. 6. The contraction ratio, which serves as the correction factor, was then calculated for each sample as the ratio of the cross-sectional area at maximum load (solid red circle) to that at zero load (solid blue circle) in Fig. 6. The range of contraction correction factors computed for all thread specimens from both the lining and the reference canvases is presented in Table 1, distinguishing between warp and weft directions. These contraction ratios will be applied in Section “Thread scale” to account for cross-sectional area reduction when calculating the *intrinsic* mechanical properties at the thread scale.
  **Fig. 6:**
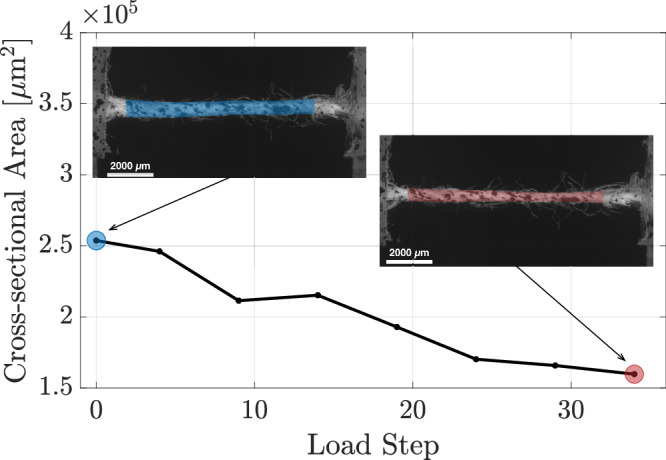
Thread scale. Evolution of the average cross-sectional area of a thread sample from the lining canvas of *The Night Watch* during loading. The initial cross-sectional area was estimated from top and lateral microscopy images, assuming an elliptical cross-section with a constant aspect ratio. During loading, changes in cross-sectional area were quantified from width variations determined from top-view microscopy images. The insets show top views at the onset of loading and at maximum load; red and blue circles indicate the averaged areas used to calculate the contraction ratio.
  **Table 1:** Thread scale

	Lining canvas		Reference canvas	
*Correction factor*	Warp	Weft	Warp	Weft
Contraction	0.52–0.92	0.74–0.95	0.56–0.97	0.65–0.73
Fibre packing density	0.86	0.86	0.86	0.86
Wax	0.64	0.68	-	-

Correction factors computed at the thread scale, which are applied to the thread cross-sectional area to calculate the *intrinsic* properties of the thread specimens in Section “Thread scale”.
- *Fibre packing density correction factor*. The fibre packing density correction factor accounts for the inherent porosity of the thread structure, as voids remain within the cross-sectional area due to the arrangement of linen fibres, even under maximum tensile load. To estimate the fibre packing density, a reference elliptical cross-sectional area of the thread was considered, with minor and major axes of 45 μm and 350 μm, respectively. These dimensions correspond to the average measured values of the minor and major axes of the thread specimens, obtained using the procedure described in Section “In-situ tensile tests on thread specimens”. Individual fibres were assumed to have circular cross-sections with an average diameter of *d* = 10 μm, as described in Section “In-situ tensile tests on fibre specimens”. The packing density of the circular fibre cross-sections within the elliptical thread was calculated based on a hexagonal lattice defined by Voronoi cells. In an infinite two-dimensional plane, this lattice achieves the highest possible circle packing density among all lattice arrangements, namely 0.907^35,36^. However, when the packing is constrained to ensure that all individual fibre cross-sections remain fully contained within the elliptical cross-section of the thread, the resulting packing density is reduced to 0.86, due to increased porosity near the thread boundary, as reported in Table 1. This packing density will be used in Section “Thread scale” as a correction factor for the cross-sectional area of all thread specimens from both the lining and reference canvases, in both warp and weft directions.
- *Wax correction factor*. A comparison of thread cross-sectional areas from the lining and reference canvases showed that, on average, the cross-sections of threads from the lining canvas are 38% larger in the warp direction and 34% larger in the weft direction than those from the reference canvas. This indicates that the presence of wax resin may result in a substantial overestimation of the load-bearing area attributed to the fibre phase. To isolate the contribution of the fibre phase, the cross-sectional area of the lining canvas threads was corrected based on the actual fibre volume fraction, thereby excluding the resin component as follows. The volume fractions of the linen fibre and wax resin components were estimated by analysing three thread samples from both the warp and weft directions of the lining canvas. Each thread was first weighed to determine the total mass, *m*_*t*_, comprising the mass of the linen fibres, *m*_*f*_, and the mass of the wax resin, *m*_*w*_, i.e., *m*_*t*_ = *m*_*f*_ + *m*_*w*_. To isolate the fibre phase, the samples were soaked for 24 h in a mixture of ethanol and acetone inside sealed vials to extract the wax resin. Subsequently, the specimens were sonicated in an ultrasonic bath inside their vials and rinsed with acetone to remove any remaining wax residue. The thread specimens were dried and conditioned at 52% RH and room temperature for 48 h to stabilise their moisture content. They were then reweighed, providing the mass ${m}_{f}^{* }$. This mass is expected to be lower than the actual fibre mass *m*_*f*_, as sonication may cause some fibre material to be lost. To quantify the fibre mass loss *m*_loss_, three control warp thread samples − taken from the reference canvas and thus free of wax resin − were first weighed, then subjected to sonication, and finally reweighed. The mass loss *m*_loss_ was calculated as the average difference between the initial and final masses of these three samples. The mass of the linen fibre phase for each thread specimen was then computed as ${m}_{f}={m}_{f}^{* }+{m}_{loss}$, from which the mass of the wax resin was obtained as *m*_*w*_ = *m*_*t*_ − *m*_*f*_. For each specimen, the volume of each constituent was determined using its respective density: for the linen fibre phase, *V*_*f*_ = *m*_*f*_/*ρ*_*f*_, where *ρ*_*f*_ = 1.5 g/cm^3^ ^37,38^ is the density of linen fibres, and for the wax resin, *V*_*w*_ = *m*_*w*_/*ρ*_*w*_, where *ρ*_*w*_ is the density of the wax resin. As discussed in Section “Characteristics of the canvas support of The Night Watch”, the wax resin used for the lining canvas of *The Night Watch* is a mixture of beeswax and colophony in an average ratio of 5:2^34^. Based on a weighted average of the densities of bee wax (density *ρ*_*bw*_ = 0.96 g/cm^3^)^39^ and colophony (density *ρ*_*p*_ = 1.07 g/cm^3^)^40^, the overall density of the wax resin was finally obtained as *ρ*_*w*_ = 0.98 g/cm^3^. After the volumes *V*_*f*_ and *V*_*w*_ were determined, the volume fractions of the linen fibre material and the wax resin were calculated as *w*_*f*_ = *V*_*f*_/(*V*_*f*_ + *V*_*w*_) and *w*_*w*_ = 1 − *w*_*f*_, respectively. The average volume fractions of the linen fibre material *w*_*f*_ in the warp and weft threads were 0.64 and 0.68, respectively, as summarised in Table 1. In Section “Thread scale”, these volume fractions will be applied to adjust the cross-sectional areas of the lining canvas thread specimens, based on the assumption that the wax resin is uniformly distributed along the thread length and does not contribute to the load-bearing capacity.

### In-situ tensile tests on canvas specimens

The specimens from the lining canvas of *The Night Watch* and the reference canvas were mechanically characterised at the canvas scale using in-situ micro-tensile testing in combination with optical microscopy.

### Sample preparation and cross-sectional area evaluation

**Fig. 7:**
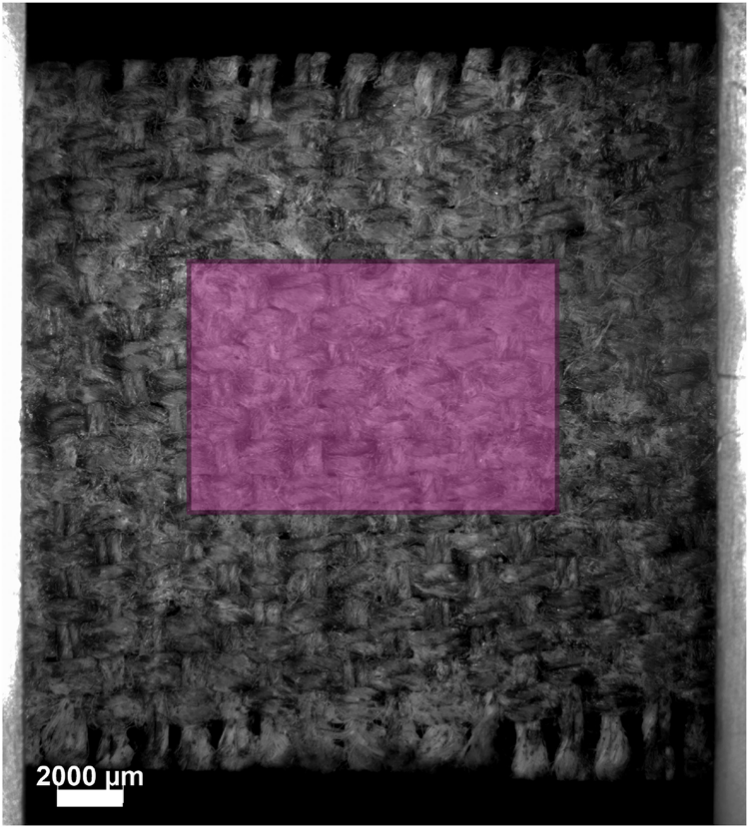
Canvas scale. Optical microscopy image of a specimen from the lining canvas of *The Night Watch*, with the warp threads oriented horizontally. The darker, slightly visible spots correspond to the applied DIC speckle pattern. The magenta rectangle indicates the ROI used for strain evaluation employing DIC. The PMMA slab or nail-polish-saturated areas are not visible because the image was acquired with the specimen mounted in the tensile stage.

During the replacement of the original stretcher of *The Night Watch* with a new aluminum strainer equipped with a spring-tensioning system^41^, four small pieces were cut from the corners of the lining canvas to facilitate its attachment to the new strainer. One of these pieces, measuring approximately 90 × 90 mm^2^, was made available for testing. From this piece, rectangular specimens were then prepared, providing four samples in both the warp and weft directions. The specimens were cut to a length of 45 mm and designed with an effective width of 20 mm for axial loading. For comparison, additional specimens with identical dimensions were prepared from the reference canvas. An area of 20 × 10 mm^2^ at each end of the specimens was designated for gripping. These areas were saturated with nail polish, then sandwiched between two 20 × 10 mm^2^ PMMA slabs and left under a weight for 24 h to ensure full curing of the adhesive. For the specimens from the lining canvas, the same method was attempted; however, the nail polish did not cure even after three days, likely due to restricted air circulation caused by the presence of wax resin. Consequently, an alternative gripping technique was developed for the specimens from the lining canvas. Instead of using PMMA slabs, two thick layers of nail polish were applied directly to the gripping areas. This modification ensured adequate gripping of the specimens in the clamps of the micro-tensile stage, see Fig. 7. Finally, a DIC speckle pattern was applied to the specimen’s top surface by spraying black paint from approximately 15 cm away under a fume hood. An example of a canvas specimen mounted in the micro-tensile stage is shown in Fig. 7, where loose transverse threads are visible along the top and bottom edges, and the darker, slightly visible spots correspond to the applied DIC speckle pattern.

**Fig. 8:**
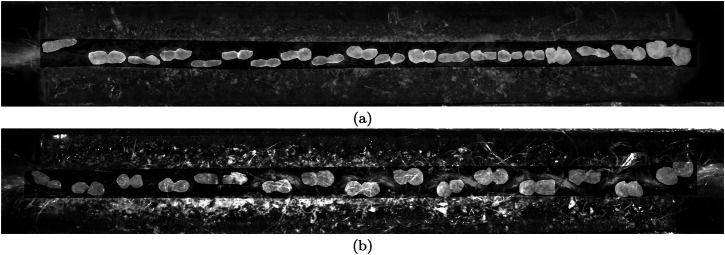
Canvas scale. Optical microscopy images of cross-sections of specimens in the **a** warp and **b** weft directions. The nominal area of each specimen is highlighted with a darker shade, while the effective cross-sectional area is given by the sum of the areas of all the threads (depicted in light grey).

The cross-sectional area of the canvas specimens was measured prior to tensile testing. Canvas specimens have a complex microstructure characterised by a highly porous cross-section. Figure 8 presents the cross-section of a canvas specimen, with Fig. 8a and b showing the warp and weft directions, respectively. The difference in thread density between these two directions is clearly visible. The effective load-bearing area, which corresponds to the sum of the thread areas depicted in light grey in Fig. 8, is significantly smaller than the nominal cross-sectional area. To accurately determine the effective load-bearing area, cross-sectional images were captured at both edges of the specimen, where the individual thread areas were measured and summed. The values from both edges were then averaged. Note that this effective cross-sectional area includes only the threads aligned with the loading direction. Since the applied loading is uniaxial, transverse threads are assumed not to carry load and are excluded from the area computation.

### Micro-tensile test

After preparing the canvas samples, the in-situ tensile tests were conducted using a Kammrath Weiss micro-tensile stage with a 1000 N load cell. Each specimen was firmly clamped to prevent slippage during loading, and images were acquired with a ZEISS Discovery V.20 stereo microscope. Optical microscopy was selected because, at this scale, profilometry was unnecessary given the minimal out-of-plane deformation of the canvas specimens. The specimens were loaded continuously under displacement control at a rate of 5 μm/s, with images acquired every 5 s until specimen failure. GDIC was employed to measure the strain in the specimens at each load step.

An ROI was selected in the centre of the specimen, see Fig. 7, thus excluding areas near the edges where complex, non-uniform deformations may occur. To account for some deformation inhomogeneity and ensure the convergence of the DIC analysis, the displacement field was parameterised using a second-order polynomial. With this choice, the axial strain field − calculated as the derivative of the displacement field in the loading direction − becomes linear. This was considered sufficiently accurate, as the strain is expected to be nearly homogeneous within the ROI. At each load step, the average axial normal strain over the ROI was computed from the axial strain field. The corresponding stress at each load step was determined by dividing the axial force, recorded by the load cell, by the previously calculated effective cross-sectional area.

### Correction factors for cross-sectional area

To determine the *intrinsic* mechanical properties at the canvas scale, the cross-sectional areas of the specimens, as deduced in Section “In-situ tensile tests on canvas specimens”, were adjusted to reflect only the load-bearing contribution of the fibre material. This was achieved by applying four correction factors. The first correction factor accounts for area contraction during loading, the second for fibre packing density, and the third for the spreading of threads caused by sample preparation. The fourth correction factor isolates the fibre volume fraction by excluding the contribution of the wax-resin matrix from the solid phase.

- *Contraction correction factor*. In canvas samples, the threads consist of loosely twisted fibres that undergo significant lateral contraction under axial loading. The contraction correction factor was computed separately for the four sample groups - lining canvas (warp, weft) and reference canvas (warp, weft) - as the mean of the thread-scale factors (listed in Table 1) from all thread specimens of each group. The resulting contraction correction factors are reported in Table 2.
  **Table 2:** Canvas scale

	Lining canvas		Reference canvas	
*Correction factor*	Warp	Weft	Warp	Weft
Contraction	0.73	0.84	0.74	0.68
Fibre packing density	0.86	0.86	0.86	0.86
Thread cutting spread	0.75	0.74	0.53	0.41
Wax	0.64	0.68	-	-

Correction factors computed at the canvas scale, which are applied to calculate the *intrinsic* properties of the canvas samples in Section “Canvas scale”.
- *Fibre packing density correction factor*. The fibre packing density correction factor used at the canvas scale was assumed to be identical to that determined at the thread scale in Section “In-situ tensile tests on thread specimens”, as listed in Table 1. For completeness, this value, which is applied to all canvas specimen types, is also included in Table 2.
- *Thread cutting spread correction factor*. During the preparation and cutting of canvas specimens, the fibres within the threads tend to spread at the cut surfaces. This localised spreading can lead to an overestimation of the load-bearing cross-sectional area, which is measured from images taken at the cut ends of the axial threads in each specimen. To correct for the effect of cutting-induced fibre spread, the average thread cross-sectional area, ${\bar{A}}_{th}$, is first retrieved for each sample type (lining and reference canvases, in both warp and weft directions), as determined from the measurements performed at the thread scale, see Section “In-situ tensile tests on thread specimens”. Note that ${\bar{A}}_{th}$ is not affected by fibre spreading, as it was determined at locations away from the cut ends, using images captured from the top and side surfaces of the threads. Next, for each canvas type, the average thread cross-sectional area including the effect of fibre spreading, ${\bar{A}}_{cn}$, is determined from images of the cut ends of the canvas specimens, as shown in Fig. 8. This is done by dividing the total thread cross-sectional area at the edges by the total number of threads (40 in the warp direction and 30 in the weft direction). These values are then averaged across all specimens of each canvas type to obtain the value for ${\bar{A}}_{cn}$. Subsequently, the cutting spread correction factor is calculated as ${\bar{A}}_{th}/{\bar{A}}_{cn}$ and is listed in Table 2 for the warp and weft directions of both the lining and reference canvases. Note that the cutting spread effect is more pronounced in the reference canvas than in the lining canvas. This is likely due to the presence of wax resin in the lining canvas, which reduces the tendency of the threads to spread at the cut sections, resulting in a smaller discrepancy between ${\bar{A}}_{th}$ and ${\bar{A}}_{cn}$.
- *Wax correction factor*. The fibre volume fraction correction factor − excluding the contribution of the wax resin − used at the canvas scale was assumed to be identical to that determined at the thread scale in Section “In-situ tensile tests on thread specimens”, as listed in Table 1. Since the specimens from the reference canvas do not contain wax resin, this correction is not applicable to these samples. For completeness, the values for the lining canvas, in both the warp and weft directions, are given in Table 2.

## Results

### Fibre scale

**Fig. 9:**
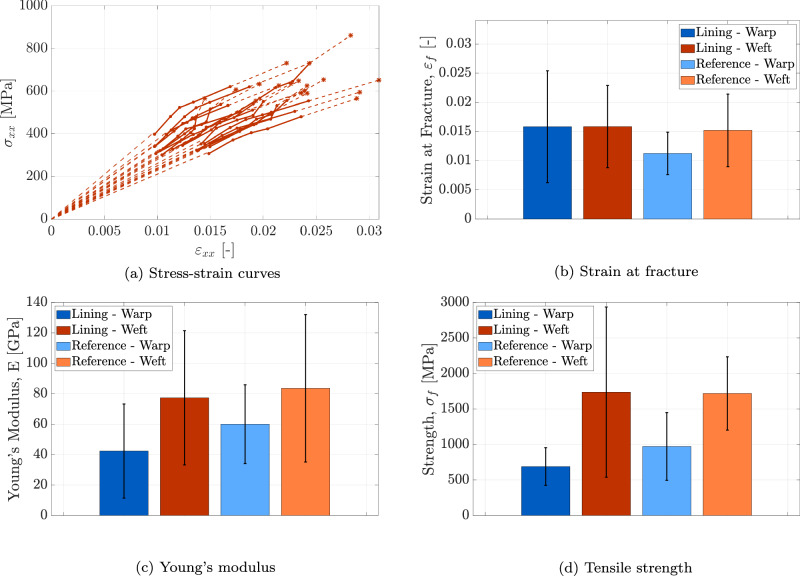
Fibre scale. Results of the micro-tensile test on fibre specimens from the lining canvas of *The Night Watch* and the reference canvas, in both warp and weft directions. **a** Local stress-strain curves evaluated from 18 ROIs along a single fibre from the weft direction of the lining canvas. **b** Mean value and standard deviation of the strain at fracture (*ε*_*f*_). **c** Mean value and standard deviation of the Young’s modulus (*E*). **d** Mean value and standard deviation of the tensile strength (*σ*_*f*_). Error bars indicate the standard deviation.

The experimental methodology presented in Section “In-situ tensile tests on fibre specimens” was applied to fibre specimens extracted from the lining canvas − 10 in the warp and 11 in the weft direction − and from the reference canvas − 4 in the warp and 6 in the weft direction. Figure 9a illustrates the stress-strain response (*σ*_*xx*_ − *ε*_*xx*_) of *one* representative fibre from the weft direction of the lining canvas, based on data collected from 18 ROIs. Each curve reflects a local mechanical response of the fibre, measured at a specific ROI. During the initial loading steps, the fibres undergo uncurling and straightening. As this stage primarily reflects geometric realignment rather than elastic deformation, these early data were excluded from the analysis. After removing these points, the initial portion of the stress-strain curve − depicted by a dashed line − was reconstructed by extrapolating the measurement data back to the origin using a first-order polynomial fit. The resulting curves exhibit an approximately linear trend up to abrupt fracture. This brittle failure behaviour may be attributed to fibre embrittlement caused by ageing processes, consistent with observations for other cellulose-based fibres^24,42–44^. Since fracture occurs abruptly after the acquisition of the final height profile, GDHC cannot capture the actual strain at fracture. Therefore, for each ROI, the strain at fracture is estimated by extrapolating a first-order polynomial fit through the last three data points of the stress-strain curve up to the final stress at fracture, calculated as the last recorded force divided by the fibre’s minimum cross-sectional area. This estimation is illustrated in Fig. 9a by the dashed line at the end of each curve, with an asterisk symbol marking the local strain at fracture. The variability in the local responses of the reference fibre is considerable, reflecting its heterogeneous nature. It is worth noting that local stresses and strains measured on a single fibre allow the deduction of statistically representative material parameters (elastic modulus, ultimate tensile strength, strain at fracture) from a relatively small number of tensile tests. Similar stress-strain curves were obtained for the other fibres tested, from both the lining and reference canvases, in warp and weft directions, which are omitted here for brevity.

Based on the stress-strain responses of all tested fibre samples, the average mechanical properties are determined. For each fibre, the strain at fracture is first calculated as the mean of the local strain at fracture values across all ROIs. These values are then averaged over all specimens within each category to obtain the final average strain at fracture, *ε*_*f*_. The corresponding standard deviation, computed across the fibre samples, is reported alongside *ε*_*f*_ in Fig. 9b. In this figure, the dark blue and red bars represent the lining canvas in the warp and weft directions, respectively, while the light blue and orange bars denote the reference canvas in the warp and weft directions, respectively. This colour scheme will be applied consistently throughout the manuscript to distinguish sample types.

The strain at fracture is broadly consistent across three of the four sample types, with mean values clustering around 0.015. A notable exception is observed for the warp-direction fibres of the reference canvas, which exhibit a significantly lower average strain at fracture of 0.011. In addition, the standard deviations for all specimen types range from 0.004 to 0.010. The average strains at fracture largely fall within the experimental range of 0.012 to 0.033 reported in the review paper ref. ^45^, which summarises data on linen (flax) fibres. However, the present measurements lean toward the lower end of this range. This difference may be attributed to the prevalent use of global strain assessment methods in previous studies (e.g.,^28,29,46^), where the strain is inferred from the relative displacement of the tensile stage grips. This approach may considerably overestimate strain values, as it also includes the effects of fibre slippage at the grips. In contrast, the present study employs a local strain measurement at the fibre level, providing a more accurate and representative evaluation of the strain at fracture.

The Young’s modulus for each ROI was calculated from the initial slope of the stress-strain curve, and then averaged across the ROIs to obtain fibre-level values. Subsequently, these were averaged per fibre type. Figure 9c shows that the average Young’s modulus *E* ranges from 42.3 to 83.6 GPa across the four fibre types. The measured values fall within the broader range of 27.6 to 103 GPa reported for flax fibres in ref. ^45^. However, it should be noted that ref. ^45^ does not distinguish between weft and warp fibre orientations. Moreover, the standard deviation of *E* ranges from 25.9 to 48.4 GPa, indicating considerable variability in Young’s modulus due to the intrinsic heterogeneity of linen fibres. Fibres oriented in the warp direction exhibit lower Young’s moduli compared to those in the weft direction. This can be explained by the fact that warp threads are typically subjected to higher tension during weaving and must remain taut on the loom afterwards. This sustained tension restricts their ability to shrink and relax when the fabric is unloaded, whereas the weft threads are free to deform more easily. The constrained shrinkage and residual stresses in the warp threads can induce micro-damage within the fibres, such as micro-cracking or fibre misalignment, ultimately leading to a reduction in their stiffness. Note further that fibres extracted from the lining canvas exhibit lower Young’s moduli compared to the corresponding fibre types from the reference canvas. This trend, observed consistently across different scales and further examined in Sections “Thread scale” and “Canvas scale”, is provisionally attributed to the more extensive degradation experienced by the lining canvas. This degradation may be hypothesised to be the result of the presence of wax-resin, the environmental display conditions of the lining canvas, and the constant stress exerted by the stretcher, since the reference canvas lacks wax-resin, was stored under controlled environmental conditions, and remained stress-free. However, the large standard deviations observed introduce significant uncertainty in this interpretation, suggesting that the mechanical properties of the lining and reference canvases may be more similar than the trend implies. The hypothesis on the influence of wax resin and display conditions on degradation will be statistically evaluated in Section “Assessing degradation: statistical comparison between the lining canvas and the reference canvas”.

The tensile strength of each fibre is determined as the ratio of the maximum applied tensile force to the smallest cross-sectional area across the fibre length. The fibre tensile strengths were averaged for each fibre type to compute the average fibre tensile strength. Figure 9d presents both the average fibre tensile strength, *σ*_*f*_, and the corresponding standard deviation for the four different fibre types. The average tensile strengths range from 689 to 1738 MPa, with standard deviations between 266 and 1199 MPa. These values are consistent with previous studies on linen fibres, as reported in ref. ^45^. Similar to the trend observed for the Young’s modulus, the fibre tensile strength is generally higher in the weft direction than in the warp direction. This anisotropy may again be attributed to a greater constrained shrinkage and residual stresses of fibres in the warp direction. When comparing the lining canvas to the reference canvas, fibres from the lining canvas exhibit lower strength in the warp direction but similar strength in the weft direction. A more detailed statistical analysis of fibre strength for both canvases is provided in Section “Assessing degradation: statistical comparison between the lining canvas and the reference canvas”.

### Thread scale

**Fig. 10:**
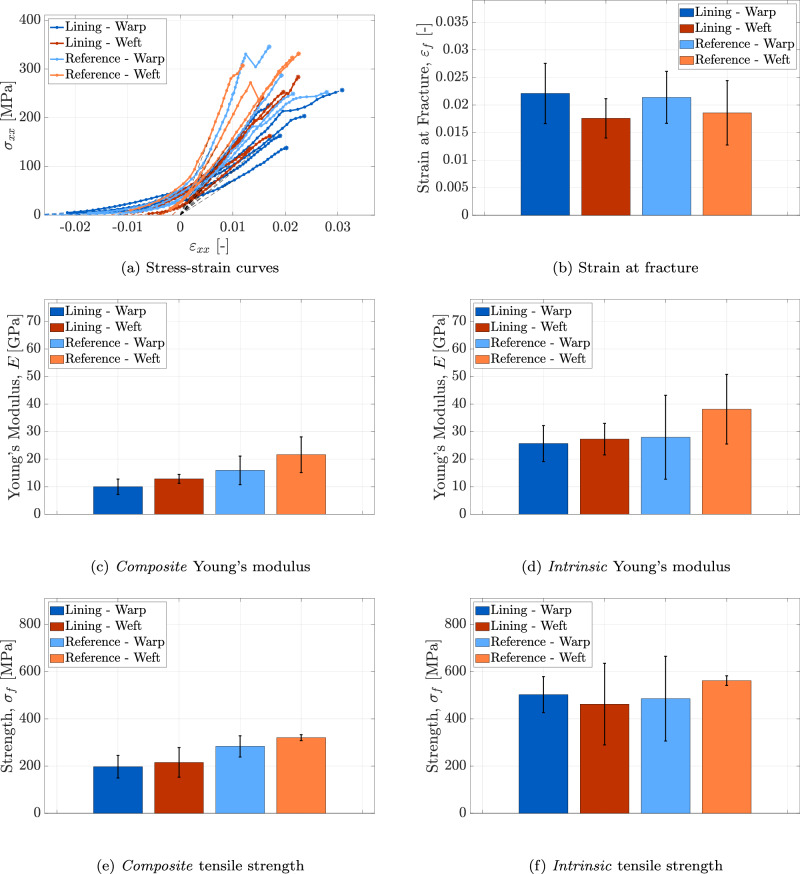
Thread scale. Results of the micro-tensile test on thread specimens from the lining canvas of *The Night Watch* and the reference canvas, in both warp and weft directions. **a** Stress-strain curves for all tested thread specimens. **b** Mean value and standard deviation of the strain at fracture (*ε*_*f*_). **c** Mean value and standard deviation of the *composite* Young’s modulus (*E*). **e** Mean value and standard deviation of the *composite* tensile strength (*σ*_*f*_). **d** Mean value and standard deviation of the *intrinsic* Young’s modulus (*E*). **e** Mean value and standard deviation of the *composite* tensile strength (*σ*_*f*_). **f** Mean value and standard deviation of the *intrinsic* tensile strength (*σ*_*f*_). Error bars indicate the standard deviation.

The experimental procedure described in Section “In-situ tensile tests on thread specimens” was applied to 5 warp and 5 weft thread samples from the lining canvas, and to 4 warp and 3 weft thread samples from the reference canvas. Hence, 17 thread specimens were tested in total, and their corresponding stress-strain (*σ*_*xx*_-*ε*_*xx*_) responses are presented in Fig. 10a. Each curve in Fig. 10a exhibits three distinct regions. In the initial region, the slope gradually increases, corresponding to the straightening of the threads under the applied load. This straightening extends over a larger deformation range in the warp threads compared to the weft threads. This behaviour is attributed to larger constrained shrinkage and residual stresses in the warp threads, ultimately leading to micro-damage within their fibres and consequently reducing their stiffness, see Fig. 9c. The reduced fibre stiffness causes the warp threads to undergo greater initial deformation than the weft threads in order to develop a given stress during straightening. Furthermore, the small initial stress measured at the onset of this region is likely caused by straightening of some of the thread’s waviness and shrinkage during the clamping process; hence, the initial part of the curve is extended to zero stress by applying a linear extrapolation (shown as a coloured dashed line) through the first two data points. The second region of the curves is characterised by an approximately constant slope, corresponding to the linear elastic response of the threads under tension. In this region, the warp threads exhibit lower elastic stiffness than the weft threads, as shown by the slope. This discrepancy reflects the effect of a higher fibre micro-damage previously described for the initial region. The third region, leading up to the maximum load, is characterised by a progressively decreasing slope, indicating the initiation of inelastic deformation due to micro-damage and/or micro-plasticity under the applied load. The extent of this region, which thus reflects progressive failure of fibres within the thread, differs among specimen types. It is noted that fibre fracture continues well beyond the point of maximum load, persisting until all fibres have failed and the tensile force in the thread reaches zero. However, the post-peak softening regime associated with progressive fibre failure is not included in the stress-strain curves depicted in Fig. 10a, as the analysis of the mechanical response beyond the peak load lies outside the scope of the present study. Furthermore, the reference for the individual stress-strain curves in Fig. 10a is established as follows: a first-order polynomial (indicated by a black dashed line) is fitted to the linear elastic portion of the curve (i.e., the second region). The entire curve is then horizontally shifted such that the fitted polynomial intersects the origin, ensuring that zero axial strain corresponds to zero axial stress. It can be observed that the stress-strain curves display substantial variability, primarily due to heterogeneities in the flax fibres and variations introduced during the flax yarn manufacturing process. Similar variability has been reported in other experimental studies^19,47^.

The strain at fracture for each thread sample is obtained from the stress-strain curves, corresponding to the (adjusted) strain at maximum load. This value is then averaged across each sample category to determine the average strain at fracture, denoted as *ε*_*f*_. Figure 10b presents the average strain at fracture along with its standard deviation, indicated by the error bars. Despite frequent imaging during testing, an image corresponding exactly to the maximum load is typically unavailable. Therefore, to estimate the strain at fracture, the same approach used for the fibre specimens in Section “Fibre scale” is applied: a first-order polynomial is fitted to the final three data points of the stress-strain curve and extrapolated to the stress value at fracture, as indicated by the dashed line at the end of each curve in Fig. 10a. However, due to the higher imaging frequency employed for thread specimens (optical images) compared to fibre specimens (surface height profiles), the extrapolated segment is very short and barely visible. No consistent trend can be identified between the strain at fracture of threads from the lining canvas and the reference canvas. Nonetheless, the strain at fracture in the warp direction seems to be larger than that in the weft direction − a characteristic not fully evident at the fibre scale. Across all specimen types, the average strain at fracture ranges between 0.018 and 0.022, with standard deviations varying from 0.004 to 0.006. The average strain values align with literature data reporting values ranging from 0.017 to 0.037 for different flax threads^18,47^.

Two separate sets of results are presented for the Young’s modulus and tensile strength, distinguishing between the *composite* and *intrinsic* properties of the thread. The strain at fracture, however, is unaffected by this distinction, as it is independent of the cross-sectional area. Figure 10c presents the mean value and standard deviation of the *composite* Young’s modulus, whereas Fig. 10d shows these values for the *intrinsic* Young’s modulus. The average *composite* Young’s modulus measured across all tested specimens falls within the range of 10.0−21.6 GPa. These values align with the range of 6.4−16.7 GPa reported in previous studies on linen (flax) yarns^19^, which do not differentiate between the warp and weft directions. The standard deviation of the *composite* Young’s modulus across all specimen types ranges from 1.6 to 6.5 GPa. This relatively large variability reflects the inherent heterogeneity at the thread scale, comparable to what is observed at the fibre level. In contrast, the average *intrinsic* Young’s modulus measured for the tested specimens ranges from 25.6 to 38.1 GPa, with standard deviations spanning from 5.7 to 15.2 GPa. For both the *composite* and *intrinsic* Young’s moduli, it is observed that threads extracted from the lining canvas tend to exhibit lower Young’s moduli compared to those from the reference canvas. While this trend could hypothetically be attributed to a higher degree of degradation in the lining canvas − possibly linked to the presence of wax resin and prolonged exposure to gallery conditions − the large standard deviation in the data, as discussed at the fibre scale in Section “Fibre scale”, indicates that the mechanical properties of the two canvases may be more similar than the apparent trend suggests. Statistical analyses presented in Section “Assessing degradation: statistical comparison between the lining canvas and the reference canvas” will further evaluate the significance of the differences observed between the lining and reference canvases. Furthermore, the properties measured in the warp direction are generally lower than those in the weft direction. This difference may again be attributed to the higher constrained shrinkage and residual stresses in the warp threads, which promote micro-damage formation. It is also important to note that the *intrinsic* Young’s moduli exhibit significantly higher values compared to the *composite* moduli. The *intrinsic* Young’s moduli represent only the load-bearing capacity of the fibre material − the stiffest constituent − while excluding the influence of the porous structure and the wax-resin. Additionally, the *composite* Young’s moduli display considerable variation among the sample groups, reflecting the heterogeneous distribution of wax resin and pores. When the cross-sectional area correction factors are applied, the resulting *intrinsic* Young’s moduli reveal a more consistent trend across the samples, with the exception of the reference canvas in the weft direction.

The strength of each thread is determined by dividing the peak load obtained from the stress-strain curves by the smallest measured cross-sectional area. The individual thread strength values are then averaged within each sample category. Figure 10e presents the average *composite* tensile strength *σ*_*f*_ along with its standard deviation, while Fig. 10f illustrates the average *intrinsic* strength values. Overall, the average *composite* strength ranges from 197 to 320 MPa, with standard deviations between 12 and 63 MPa. These average strength values are consistent with the reported range of 115–339 MPa for flax threads, presented in refs. ^18,19^. The average *composite* strength measured in the warp direction is generally lower than that in the weft direction. This difference may be attributed to the greater micro-damage in the warp threads, which reduces their effective load-bearing capacity. Furthermore, the strength values measured from the lining canvas samples are lower than those obtained from the reference canvas samples. These observations broadly align with trends identified at the fibre scale and will be examined in detail through statistical analyses in Section “Assessing degradation: statistical comparison between the lining canvas and the reference canvas”. For all tested specimens, the average *intrinsic* strength ranges from 462 to 562 MPa, with standard deviations spanning from 21 to 179 MPa. After applying cross-sectional correction factors, the *intrinsic* strength values are significantly higher than the corresponding *composite* strength values. Nonetheless, no consistent pattern can be identified between the warp/weft characteristics and the properties of the lining and reference canvases.

**Fig. 11:**
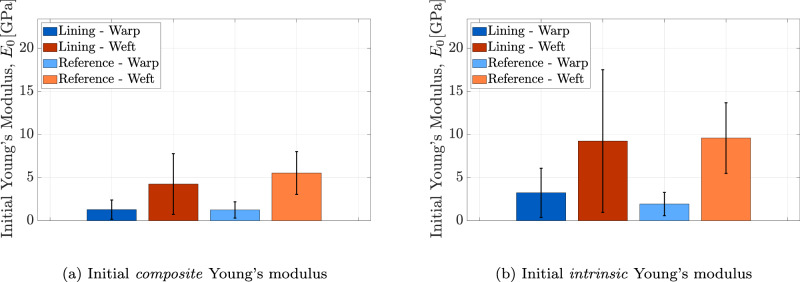
Thread scale. Results of the micro-tensile test on thread specimens from the lining canvas of *The Night Watch* and the reference canvas, in both warp and weft directions. **a** Mean value and standard deviation of the initial *composite* Young’s modulus (*E*_0_). **b** Mean value and standard deviation of the initial *intrinsic* Young’s modulus (*E*_0_). The error bars indicate the standard deviation.

The Young’s moduli presented in Fig. 10 are derived from the linear branch of the stress-strain curves shown in Fig. 10a, corresponding to the second phase following the straightening of the thread samples. However, the initial part of the stress-strain curves, where the slope gradually increases, is also of interest in this study. This region offers insight into the thread response under relatively low stress levels, which more closely represents the typical loading conditions experienced by the lining canvas in museum environments. The initial Young’s modulus *E*_0_ is determined as the slope of the first-order polynomial fitted to the first two data points of each stress-strain curve, as described previously. Figure 11 illustrates the initial Young’s modulus *E*_0_ for thread specimens from both the lining and reference canvases, in the warp and weft directions, considering *composite* and *intrinsic* properties. The average initial *composite* Young’s modulus measured across all tested specimens ranges from 1.2 to 5.5 GPa, with standard deviations between 0.9 and 3.5 GPa. In contrast, the average initial *intrinsic* Young’s modulus ranges from 1.9 to 9.6 GPa, with standard deviations between 1.4 and 8.3 GPa. For both types of initial Young’s moduli, the values in the warp direction are significantly lower than those in the weft direction, which can again be ascribed to the larger micro-damage in the warp threads. Furthermore, comparing the lining and reference canvases reveals no obvious distinction. The substantial standard deviations observed in the initial Young’s moduli of the thread specimens can likely be attributed to the inherent heterogeneity in local fibre alignment and fibre properties, see also Fig. 9.

### Canvas scale

**Fig. 12:**
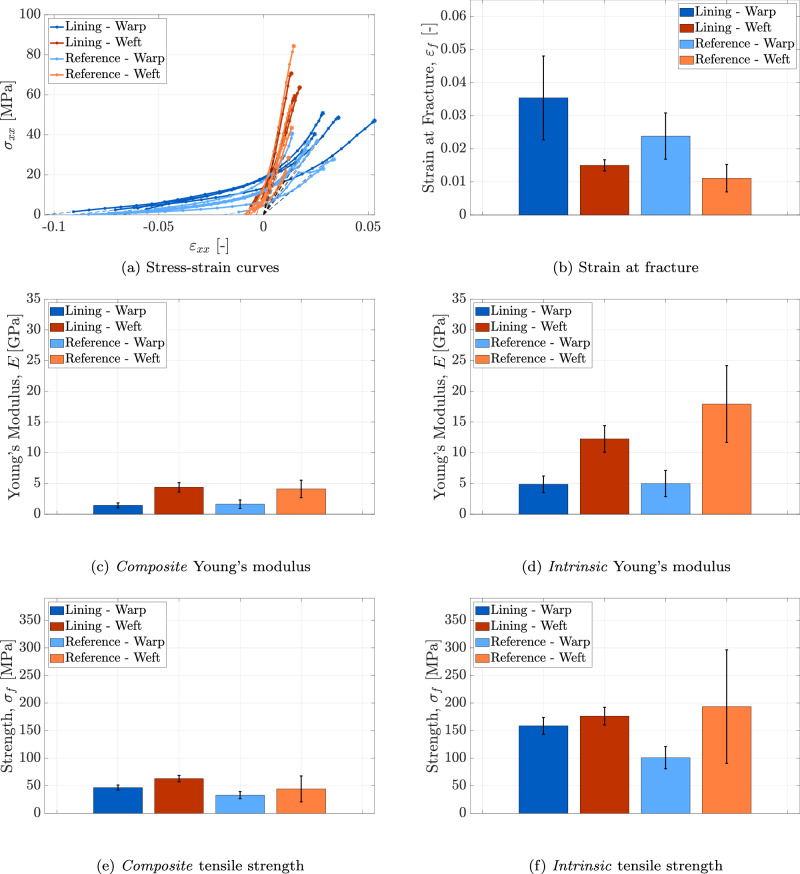
Canvas scale. Results of the micro-tensile test on canvas specimens from the lining canvas of *The Night Watch* and the reference canvas, in both warp and weft directions. **a** Stress-strain curves for all tested canvas specimens. **b** Mean value and standard deviation of the strain at fracture (*ε*_*f*_). **c** Mean value and standard deviation of the *composite* Young’s modulus (*E*). **e** Mean value and standard deviation of the *composite* tensile strength (*σ*_*f*_). **d** Mean value and standard deviation of the *intrinsic* Young’s modulus (*E*). **e** Mean value and standard deviation of the *composite* tensile strength (*σ*_*f*_). **f** Mean value and standard deviation of the *intrinsic* tensile strength (*σ*_*f*_). Error bars indicate the standard deviation.

The experimental procedure described in Section “In-situ tensile tests on canvas specimens” was applied to 4 specimens each from the warp and weft directions of the lining canvas, and 6 specimens each from the warp and weft directions of the reference canvas. Figure 12a shows the resulting stress-strain curves (*σ*_*xx*_-*ε*_*xx*_) for the 20 canvas samples. Similarly to the thread response in Fig. 10a, these curves display an initial nonlinear region, an approximately linear elastic region, and a post-yield region leading to catastrophic failure. Consistent with the thread scale, the curves have been horizontally shifted so that the extrapolation of the linear elastic branch (black dashed line) runs through the origin of the diagram. Further, the initial part of each stress-strain curve is linearly extrapolated to zero stress using the first two data points of the curve (coloured dashed line). For reasons similar to those discussed in Section “Thread scale” with respect to the thread responses presented in Fig. 10a, canvas specimens loaded in the warp direction exhibit greater deformation during the initial straightening phase (first region) and display lower elastic stiffness in the subsequent linear elastic regime (second region), compared to those loaded in the weft direction.

The strain at fracture for each canvas specimen is determined from the stress-strain curves at the point of maximum load. The average strain at fracture, *ε*_*f*_, together with its standard deviation, is presented in Fig. 12b. These results indicate that specimens loaded in the warp direction exhibit a higher strain at fracture than those loaded in the weft direction. This observation is consistent with the thread-scale behaviour reported in Section “Thread scale”. For all specimen types, the average fracture strain ranges from 0.011 to 0.035 with standard deviations between 0.002 and 0.013, with the lining canvas specimens showing higher fracture strain values in both the warp and weft directions compared to the reference canvas. It is worth noting that the strain at fracture values reported in previous studies on linen-based canvas paintings (e.g., ref. ^8^) are significantly higher − approximately 0.25 for the warp direction and 0.13 for the weft direction. However, in ref. ^8^, the strain at fracture was calculated by *including* the initial strain associated with the straightening phase of the threads, and should therefore be compared to the maximum, total strain values measured in the current study. As can be observed from Fig. 12a, for the lining canvas in the warp direction the total strain reaches a maximum of approximately 0.15. Although this total strain value is closer to that reported in ref. ^8^, it remains lower, likely due to differences in the materials, strain measurement methods, and evaluation criteria employed in each study. In particular, the disparity in strain measurement approaches plays a significant role: the current study uses local strain evaluation at the canvas scale, whereas ref. ^8^ relies on global strain measurements that tend to overestimate strain, see also the discussion in Section “Fibre scale”.

Following the same approach applied at the thread scale, the composite and intrinsic Young’s moduli and tensile strengths are evaluated. Figure 12c shows the mean value and standard deviation of the *composite* Young’s modulus *E* for the four canvas types, whereas Fig. 12d displays the corresponding values for the *intrinsic* Young’s modulus. The mean values of the *composite* Young’s modulus measured across all specimens range from 1.43 to 4.37 GPa, with standard deviations between 0.40 and 1.43 GPa. These values are higher than those reported in previous studies investigating the mechanical properties of linen canvases ^1,2,8^. Note, however, that in these earlier works the reported *elastic modulus* corresponds to the *initial modulus* derived from the initial slope of the stress-strain curve, whereas in the present analysis the modulus corresponds to the subsequent, approximately linear region. This difference in definition accounts for the higher values obtained here. The mean values of the *intrinsic* Young’s modulus range between 4.86 and 17.91 GPa, with standard deviations from 1.35 to 6.26 GPa. For both the *composite* and *intrinsic* Young’s moduli, specimens loaded in the warp direction consistently show lower mean values than those loaded in the weft direction. This trend again reflects the effect of larger micro-damage in the warp fibres. Overall, the lining canvas specimens generally show Young’s moduli that are similar to or slightly lower than those of the reference canvas; a statistical analysis quantifying these variations is presented in Section “Assessing degradation: statistical comparison between the lining canvas and the reference canvas”. As expected, the *intrinsic* Young’s moduli are considerably larger than the *composite* values, and the relative differences between the warp and weft directions are more pronounced at the canvas scale than at the thread scale (Fig. 10d).

Figure 12e presents the mean and standard deviation of the *composite* tensile strength *σ*_*f*_ for the four canvas types, while Fig. 12f shows the corresponding *intrinsic* tensile strength values. Overall, the mean *composite* strength ranges from 33 to 63 MPa, with standard deviations between 4 and 24 MPa, which aligns with the measurements reported in ref. ^2^, when differences in specimen width are taken into account. The mean value of the *intrinsic* ultimate tensile strength across all canvas types ranges from 101 to 193 MPa, with standard deviations between 15 and 103 MPa. Both the *composite* and *intrinsic* tensile strengths are generally lower in the warp direction compared to the weft direction. Interestingly, unlike the trends observed on the fibre and thread scales, the *composite* tensile strengths of the lining canvas in warp and weft directions exceed those of the reference canvas, whereas the *intrinsic* tensile strength does not exhibit any clear or consistent difference between the two.

**Fig. 13:**
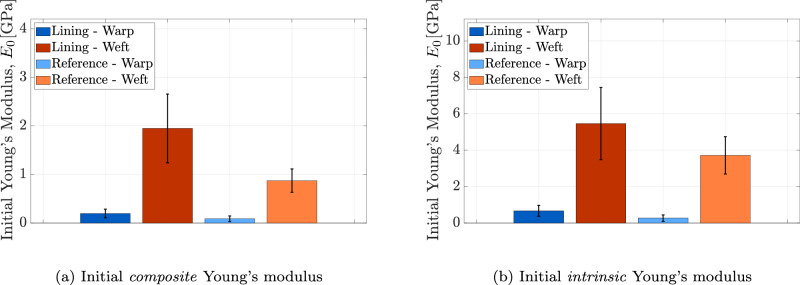
Canvas scale. Results of the micro-tensile test on canvas specimens from the lining canvas of *The Night Watch* and the reference canvas, in both warp and weft directions. **a** Mean value and standard deviation of the initial *composite* Young’s modulus (*E*_0_). **b** Mean value and standard deviation of the initial *intrinsic* Young’s modulus (*E*_0_). The error bars indicate the standard deviation.

Figure 13 shows the initial Young’s modulus *E*_0_ measured at the onset of loading for the lining and reference canvases, in both warp and weft directions, with results reported for *composite* and *intrinsic* properties. As part of the recent retensioning procedure carried out during *Operation Night Watch*, the newly installed spring tension system applied carefully controlled loads per unit length of 2.7 N/cm and 2.0 N/cm in the warp and weft directions, respectively^41^. These loads correspond to stresses below 1 MPa on the lining canvas, calculated based on its thickness and *composite* properties. Determining the initial Young’s modulus *E*_0_ is therefore particularly relevant, as it characterizes the canvas stiffness under these low in-situ stress levels applied by the tensioning system. Observe from Fig. 13 that, consistent with observations at the thread scale, the initial Young’s modulus *E*_0_ in the warp direction is lower than in the weft direction. In ref. ^8^, the reported (initial) Young’s moduli range from 0.05 to 0.50 GPa for the warp direction and from 0.2 to 0.9 GPa for the weft direction. By comparison, the *composite* initial Young’s modulus determined in this study varies between 0.06 and 0.22 GPa for the warp direction and between 0.79 and 2.91 GPa for the weft direction. These values fall within the same order of magnitude but are modestly higher than those reported by ref. ^8^. This difference may be due to the use of local strain measurements in the present study, as opposed to the global strain evaluation applied in ref. ^8^, which tends to overestimate strain and thus underestimate the (initial) Young’s modulus. The discrepancy may also simply reflect variations in sample type, including microstructure, conditions, past conservation treatments, or manufacturing.

### Scaling laws

To compare the mechanical response of the lining canvas across different structural levels, the material properties reported in Sections “Fibre scale”, “Thread scale”, and “Canvas scale” − corresponding to fibre, thread, and canvas measurements − are examined as a function of their characteristic length scales. For fibres and threads, the characteristic length scale *l*_*c*_ is defined as the square root of the average cross-sectional area, ranging from 5.6–18.2 μm to 285.7–549.7 μm, respectively. Canvas specimens, which have a basket weave formed by interwoven thread pairs, are idealised as a periodic structure. Hence, the characteristic length scale *l*_*c*_ is defined as the distance between the centres of adjacent thread pairs, calculated by dividing the specimen width (20 mm) by the number of thread pairs. Based on measured thread densities of 19 threads/cm in the warp direction and 17 threads/cm in the weft direction (see also Section “Characteristics of the canvas support of The Night Watch”), this yields characteristic length scales of approximately 1 mm for the warp direction and 1.33 mm for the weft direction.

**Fig. 14:**
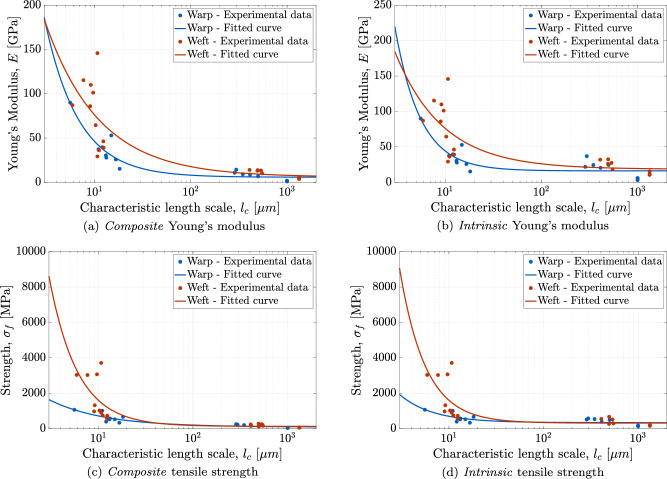
Scaling laws. Mechanical properties obtained from micro-tensile tests on fibre, thread, and canvas specimens from the lining canvas of The Night Watch, plotted as a function of the characteristic length scale *l*_*c*_, together with the fitted scaling laws given by Eq. (1). The calibrated parameters of the scaling laws are listed in Table 3. **a** *Composite* Young’s modulus (*E*) in the warp and weft directions. **b** *Intrinsic* Young’s modulus (*E*) in the warp and weft directions. **c** *Composite* tensile strength (*σ*_*f*_) in the warp and weft directions. **d** *Intrinsic* tensile strength (*σ*_*f*_) in the warp and weft directions.

Figure 14 summarises the measured mechanical properties of the lining canvas as a function of the characteristic length scale, *l*_*c*_ [*μ*m]. Blue and red markers correspond to the warp and weft directions, respectively. A logarithmic horizontal axis is used to capture the wide range of length scales. Figure 14a and b show the *composite* and *intrinsic* Young’s modulus *E*, respectively, while Fig. 14c and d present the corresponding *composite* and *intrinsic* ultimate tensile strength *σ*_*f*_. The strain at fracture does not exhibit a clear trend with length scale and is therefore omitted. Scaling laws relating the mechanical properties to the characteristic length scale *l*_*c*_ are derived from the data in Fig. 14, with the fitted relationships shown as continuous lines in each figure. The general form of the scaling law of the lining canvas is given by

1 $$M({l}_{c})=a\,{({l}_{c})}^{b}+c\,,$$

where *M* ∈ {*E*, *σ*_*f*_} denotes the evaluated material parameter (Young’s modulus or tensile strength), and *a*, *b* and *c* are calibration parameters. The values of the calibration parameters, corresponding to the Young’s modulus and tensile strength in both the warp and weft directions, along with the associated *R*^2^ values, are listed in Table 3.

**Table 3:** Scaling laws

	Young’s modulus *E* [GPa]				Tensile strength *σ*_*f*_ [MPa]			
	*a*	*b*	*c*	*R*^2^	*a*	*b*	*c*	*R*^2^
Warp - Composite properties	704	−1.24	5.8	0.90	3532	−0.75	92	0.83
Weft - Composite properties	417	−0.77	5.9	0.68	41820	−1.45	129	0.62
Warp - Intrinsic properties	1235	−1.64	16.2	0.75	6188	−1.24	341	0.54
Weft - Intrinsic properties	434	−0.88	18.4	0.60	50298	−1.59	315	0.57

Calibrated parameters of the scaling law, Eq. (1), for the Young’s modulus *E* and tensile strength *σ*_*f*_ of the lining canvas of *The Night Watch* in both warp and weft directions, referring to *composite* and *intrinsic* material properties.

Figure 14 shows that the Young’s modulus and tensile strength decrease with increasing length scale in both warp and weft directions. This reduction at larger scales is attributed to an increased probability of defects within the fibres, as well as microstructural mechanisms − such as frictional interactions and slippage between fibres − that have a more prominent cumulative effect at larger scales, collectively reducing the average stiffness and tensile strength. For the *composite* properties, the fitted scaling laws result in *R*^2^ values ranging from 0.62 to 0.90, indicating moderate to strong agreement with the experimental data. In contrast, for the *intrinsic* properties, the *R*^2^ values fall between 0.54 and 0.75, suggesting that the scaling laws capture the overall trends but with more scatter and lower predictive accuracy.

The presented scaling laws may enable, in the future, informed predictions of the mechanical properties of the lining canvas at the canvas scale, based on non-invasive fibre-level analyses rather than destructive sampling at thread or canvas scale, which is an important advantage given the limited availability of lining canvas specimens. The scaling laws for *composite* properties are directly relevant to conservation efforts for *The Night Watch*, as they reflect its specific porosity and wax-resin impregnation. In contrast, scaling laws for *intrinsic* properties are expected to show greater generality, as they relate to the ideally uniform linen fibre phase. Beyond this case study, these scaling laws may also support the analysis of other historic (lining) canvases made of linen or comparable materials, broadening their applicability to conservation strategies in cultural heritage. Finally, it is noted that similar scaling laws were derived for the reference canvas, but these are not included here for brevity.

### Assessing degradation: statistical comparison between the lining canvas and the reference canvas

The state of degradation of the lining canvas of *The Night Watch* is assessed by statistically comparing its mechanical properties with those of a reference canvas. The reference canvas, preserved in a controlled environment and free from wax-resin impregnation, serves as a benchmark to evaluate the potential effects of the Rijksmuseum gallery environment, the presence of wax-resin, and the continuous tensile stress applied to the lining canvas on its mechanical degradation. The comparison is performed across different length scales (fibres, threads, and canvas) and focuses on the *intrinsic* Young’s modulus, *intrinsic* tensile strength, and strain at fracture. Using *intrinsic* properties ensures a consistent basis for comparison by reducing the influence of confounding factors such as wax resin content, porosity, and cutting spread of threads.

To assess differences between the two data groups, an independent two-sample Student’s *t*-test is performed^48^. This test evaluates whether the means of the two groups differ significantly, under the null hypothesis that the group means are equal, within a certain statistical significance. The *t*-statistic is calculated as

2 $$t=\frac{{\overline{X}}_{1}-{\overline{X}}_{2}}{\sqrt{\frac{{s}_{1}^{2}}{{n}_{1}}+\frac{{s}_{2}^{2}}{{n}_{2}}}},$$

where ${\overline{X}}_{1},{\overline{X}}_{2}$ are the means on the specific sample quantities analysed, *s*_1_, *s*_2_ are the sample variances, and *n*_1_, *n*_2_ are the sample sizes. The *t*-value obtained through Eq. (2) for the different properties analysed is compared with a critical threshold, *t*_critical_, which depends on the selected significance level *α* and the degrees of freedom of the problem (*n*_1_ + *n*_2_ − 2). If ∣*t*∣ < *t*_critical_, the null hypothesis of equal mean values cannot be rejected. The results of the *t*-test for *α* = 0.05 are reported in Table 4.

**Table 4:** Statistical comparison between the lining canvas and the reference canvas

		Fibre			Thread			Canvas		
		E	*σ*_*f*_	*ε*_*f*_	E	*σ*_*f*_	*ε*_*f*_	E	*σ*_*f*_	*ε*_*f*_
Warp	Lining canvas samples (*n*_1_)	8	10	8	5	5	5	4	4	4
	Reference canvas samples (*n*_2_)	4	4	4	4	4	4	6	6	6
	*t* − test	*✓*	*✓*	*✓*	*✓*	*✓*	*✓*	*✓*	✗	*✓*
	*U* − test	*✓*	*✓*	*✓*	*✓*	*✓*	*✓*	*✓*	✗	*✓*
Weft	Lining canvas samples (*n*_1_)	11	11	11	5	5	5	4	4	4
	Reference canvas samples (*n*_2_)	6	6	6	3	3	3	6	6	6
	*t* − test	*✓*	*✓*	*✓*	*✓*	*✓*	*✓*	*✓*	*✓*	*✓*
	*U* − test	*✓*	*✓*	*✓*	*✓*	*✓*	*✓*	*✓*	*✓*	*✓*

Results of Student’s *t* − test and Mann−Whitney *U*-test (significance level *α* = 0.05), comparing the *intrinsic* Young’s modulus (*E*), *intrinsic* strength (*σ*_*f*_), and strain at fracture (*ϵ*_*f*_) of the lining canvas and the reference canvas in both warp and weft directions. Check marks and crosses indicate whether the null hypothesis is accepted or rejected, respectively. The sample size for each group (*n*_1_ and *n*_2_) is also reported.

The validity of this test relies on three key assumptions: (i) the samples are independent; (ii) the data approximately follow a normal distribution; and (iii) the two groups have similar standard deviations. In the present analysis, data independence is satisfied. Due to the small sample sizes, the normality assumption cannot be formally tested and is assumed a priori^49^. The assumption of comparable standard deviations − often considered reasonable if their ratio does not exceed approximately two^49^ − is met for most of the properties tested. Nevertheless, given the limited sample size, the robustness of this comparison may be limited.

To address this limitation, a Mann−Whitney *U*-test is additionally performed as an alternative statistical analysis^50^. Unlike the *t*-test, the Mann−Whitney *U*-test is non-parametric and therefore does not assume a normal data distribution or require comparable standard deviations. The test ranks all data points, where the rank of a data point corresponds to its position in the combined data set sorted in ascending order. The test calculates the *U*-statistics for the two sample groups as

3 $${U}_{1}={n}_{1}{n}_{2}+\frac{{n}_{1}({n}_{1}+1)}{2}-{R}_{1}\,{\mathrm{and}} \,{U}_{2}={n}_{1}{n}_{2}+\frac{{n}_{2}({n}_{2}+1)}{2}-{R}_{2},$$

where *R*_1_ and *R*_2_ are the rank sums of the respective groups. The smaller value between *U*_1_ and *U*_2_ is compared to the critical value *Z*_*critical*_, derived from the Mann−Whitney *U* distribution tables for the given sample sizes (*n*_1_, *n*_2_) and significance level (*α*). If ∣*U* ∣ < *Z*_critical_, the null hypothesis that the distributions are identical cannot be rejected. The results of the *U*-test for a significance level *α* = 0.05 are also reported in Table 4.

The results of both tests are consistent, with all cases − except one − supporting the null hypothesis. The exception is the tensile strength of the canvas in the warp direction, where a significant difference is observed between the means of the two sample groups. For all other cases, acceptance of the null hypothesis suggests that environmental conditions and the presence of wax resin may have had a *negligible effect* on the mechanical degradation of the lining canvas. The agreement between the *t*- and *U*-tests further strengthens the reliability of this conclusion. Although definitive statements are limited by the small sample size, this statistical analysis provides valuable insight into identifying or excluding factors that potentially contribute to canvas degradation.

## Discussion

The present study focuses on the experimental multi-scale analysis of the mechanical response of the lining canvas of *The Night Watch* by Rembrandt van Rijn. Using advanced in-situ micro-tensile testing, combined with optical microscopy, optical profilometry, and full-field deformation measurements (GDIC and GDHC), the mechanical properties − Young’s modulus, tensile strength, and strain at fracture − are characterised across three scales: fibre, thread, and canvas scales. These measurements capture the multi-scale mechanical response of the lining canvas as a *composite* structure, accounting for the contributions of fibres, wax resin, and void phases. In addition, the *intrinsic* properties, which isolate the load-bearing role of the cellulosic fibres, have been deduced from the experimental data by applying correction factors. These factors correct for cross-sectional area reduction under load, fibre volume fraction, fibre packing density, and cutting spread of threads. The main findings are summarised as follows:

- Across all scales, the *composite* and *intrinsic* properties show that the Young’s modulus and tensile strength are lower in the warp than in the weft direction. This is attributed to greater constrained shrinkage and residual stress in the warp threads, leading to fibre misalignment, distortions, and micro-damage that degrade the response across scales.
- The measured mechanical properties fall within the range of values reported in the literature. However, local strain evaluation at the fibre and canvas scales yielded values toward the lower bound of reported strains and correspondingly higher stiffness, reflecting the sensitivity of local measurements to microstructural heterogeneity.
- Scaling laws demonstrate an inverse relationship between mechanical properties (Young’s modulus, tensile strength) and characteristic length scale. The reduction in properties at larger length scales is attributed to the higher probability of defects and micro-structural mechanisms (e.g., fibre slippage), which lower stiffness and failure resistance. These scaling laws may provide reliable, minimally invasive predictions of canvas-scale properties from fibre-scale measurements.
- Samples from the lining canvas appear to exhibit lower mechanical properties than those from the reference canvas, which was initially attributed to degradation caused by the applied tensile stress, wax-resin impregnation, and prolonged display conditions. However, statistical analyses did not reveal significant differences between the two canvases, suggesting that these factors did not play a dominant role in the mechanical degradation of the lining canvas.

## Data Availability

Data will be made available on request.
